# A pilot randomised controlled parallel arm trial evaluating treatment satisfaction with the Omnipod DASH^®^ Insulin Management System compared with usual care in adults with type 1 diabetes in Australia: rationale, study design and methodologies

**DOI:** 10.1186/s40814-023-01400-4

**Published:** 2023-10-09

**Authors:** Yee Wen Kong, Cheng Yi Yuan, Katerina Kiburg, Katrin Brown, Steven Trawley, Andi Partovi, Kerryn Roem, Natalie Harrison, Spiros Fourlanos, Elif I. Ekinci, David N. O’Neal

**Affiliations:** 1 Diabetes Technology Research Group, The University of Melbourne, Melbourne, VIC Australia; 2 Department of Endocrinology and Diabetes, St Vincent’s Hospital Melbourne, Melbourne, VIC Australia; 3 Department of Medicine, The University of Melbourne, 4th Floor, Clinical Sciences Building, 29 Regent Street, Fitzroy, VIC Australia; 4 Cairnmillar Institute, Camberwell, VIC Australia; 5 Keylead Health, Melbourne, VIC Australia; 6 Geelong Endocrinology and Diabetes, Geelong, VIC Australia; 7 Department of Diabetes and Endocrinology, The Royal Melbourne Hospital, Melbourne, VIC Australia; 8 Department of Endocrinology, Austin Health, Melbourne, VIC Australia; 9 The Australian Centre for Accelerating Diabetes Innovations (ACADI), The University of Melbourne, Melbourne, VIC Australia

**Keywords:** Type 1 diabetes, Patch pump, Insulin pump therapy, Treatment satisfaction

## Abstract

**Background:**

Insulin pump therapy (IPT) improves glucose control in people with type 1 diabetes (T1D) compared with multiple daily injections (MDI). However, their size, the tethered insulin infusion set, intrusiveness when operating the device and the need to disconnect during showering limit their acceptance to many who may benefit. The Omnipod DASH^®^ Insulin Management System is a small waterproof tubeless device which is wirelessly controlled by a handheld device which may be an acceptable alternative. However, there are no randomised controlled trials focusing on the impact on user perceptions of tubeless insulin pump therapy. This pilot study aims to assess study feasibility and acceptability of patch pump therapy compared with usual care in adults with T1D in Australia to inform power calculations and progression to a large-scale multi-site randomised controlled study.

**Methods:**

A pilot multi-site parallel randomised controlled study will be conducted in sixty-four adults with T1D who are managed on MDI or IPT and self-monitoring with finger-stick blood glucose from four specialist diabetes centres in Victoria, Australia. Following carbohydrate counting education, participants will be randomised to use Omnipod DASH^®^ System (Omnipod group) or continue usual care (usual care group) for 12 weeks, followed by a 12-week extension phase where all participants will use Omnipod DASH^®^ System. The primary outcome measure is feasibility determined by study completion rates with a threshold of 0.80. Acceptability of the intervention (Omnipod DASH^®^ System) will be assessed by the difference in Diabetes Technology Questionnaire ‘current’ (DTQ-current) score at 12 weeks post-randomisation compared to baseline. Secondary outcomes will include other measures of user acceptance, process outcomes, resource outcomes, participant-centred outcomes, healthcare professional perceptions and glycaemic outcomes.

**Discussion:**

This pilot study will provide insights regarding the feasibility of the study design and the first data regarding user acceptance of insulin patch pump technology in Australian T1D adults. We anticipate that this study will provide information informing the design of a larger study evaluating the impact of patch pumps on subjective outcomes that are of significance to the person living with T1D.

**Trial registration:**

Australian New Zealand Clinical Trials Registry (https://anzctr.org.au/) ACTRN12621001195842 (8th September 2021). Please refer to Additional file 1: Appendix 1 for full details.

**Supplementary Information:**

The online version contains supplementary material available at 10.1186/s40814-023-01400-4.

## Background and rationale

Continuous subcutaneous insulin infusion or insulin pump therapy (IPT) can improve glycaemic management and the quality of life of people living with type 1 diabetes (T1D) compared with multiple daily injections (MDI) [1–3]. The conventional insulin pump is a pager-sized electronic device that delivers insulin from the reservoir through an infusion set, consisting of tubing connected to a subcutaneously inserted cannula [4].

However, despite the potential benefits, a significant subset of people with T1D choose to continue MDI even when financial barriers to IPT access are absent. This may be related to the physical inconveniences of conventional insulin pumps, which have been reported to deter IPT acceptance and increase diabetes distress [1]. The bulky size, heavy weight and presence of tubing integral to a conventional insulin pump can make wearing and operating the device discretely a challenge [4]. Moreover, the tubing can get caught on objects, causing discomfort and interruption of insulin delivery. Conventional insulin pumps are also not waterproof and need to be removed when showering or swimming to avoid damage to these expensive devices.

**Fig. 1:**
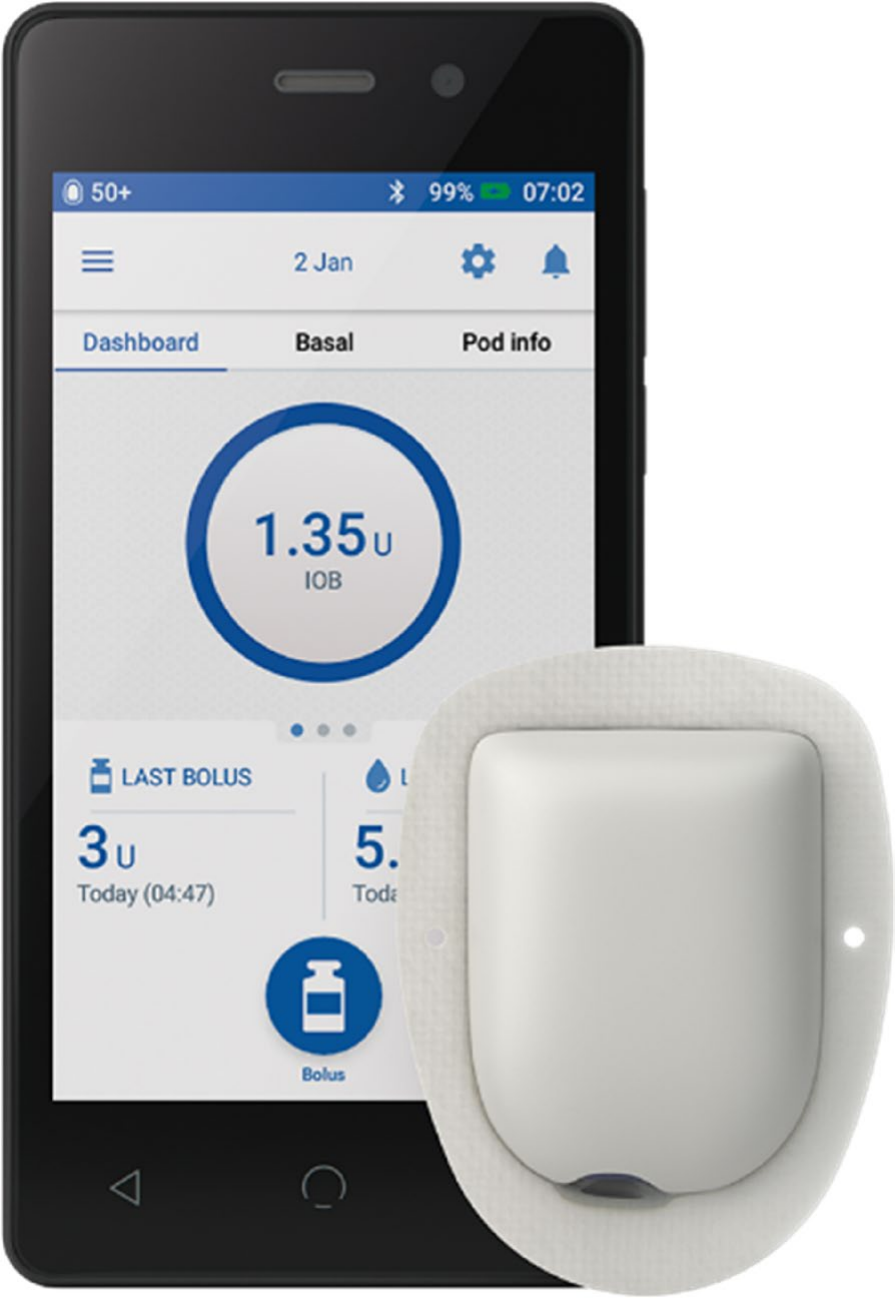
Omnipod DASH^®^ Insulin Management System (pod and Personal Diabetes Manager [PDM])

Insulin patch pumps are compact and light tubeless devices attached directly to the skin and could address many of the limitations associated with a conventional insulin pump impacting acceptance by the person living with T1D. Insulet was the first company to bring a patch pump to market and their devices are the most widely used in this category. The Omnipod DASH^®^ Insulin Management System (Acton, MA) is a small and light device (5.2cm by 3.9cm by 1.45 cm, weighing 26g) that is tubeless and attached directly onto the skin, minimising intrusiveness and allowing for freedom of movement [5]. Moreover, insulin settings are programmed via a remote handset, the Personal Diabetes Manager (PDM) which enables discreet insulin bolus delivery [5]. It is also waterproof for up to 25 feet for 60 min so it can be worn while showering or swimming. The PDM is not waterproof (Fig. 1).

Insulin patch pumps are commercially available in the most advantaged countries. Despite evidence that these devices can improve glycaemia [6], there has been no randomised controlled trial with its primary focus on the impact of tubeless insulin pump therapy on the subjective experiences of adults with T1D compared with usual care, and psychological outcomes, which include treatment satisfaction, have received less attention. Nevertheless, these outcomes are at least as important, if not more so, to the person living with diabetes and may impact the uptake of technology and the way in which the devices are used.

Our main objective is to conduct a pilot study testing the feasibility of a protocol employing a change in the Diabetes Technology Questionnaire ‘current’ (DTQ-current) score as a tool to examine the user acceptance of insulin patch pumps in adults living with T1D in comparison with usual care, defined as insulin delivery either with MDI or a conventional insulin pump in conjunction with self-monitoring of blood glucose (SMBG). This pilot study will provide feasibility data for completion rates and acceptability of insulin patch pumps to inform power calculations and progression to future large-scale trials examining the efficacy of patch pumps in improving the quality of life for adults with T1D. We will also explore other methods for assessing user acceptance (e.g. DTQ-change scores). Additional secondary process outcomes (e.g. clarity and suitability of eligibility criteria) and resource outcomes (e.g. determining the functionality of electronic data collection tools such as the KeyLead™ application) that will be key to the successful implementation of a definitive large randomised control study aimed at evaluating the user acceptance of patch pump insulin delivery will also be assessed.

Secondary scientific aims include the evaluation of participant-reported outcomes and healthcare professional perspectives on insulin patch pump therapy, and metrics related to glucose control.

**Fig. 2:**
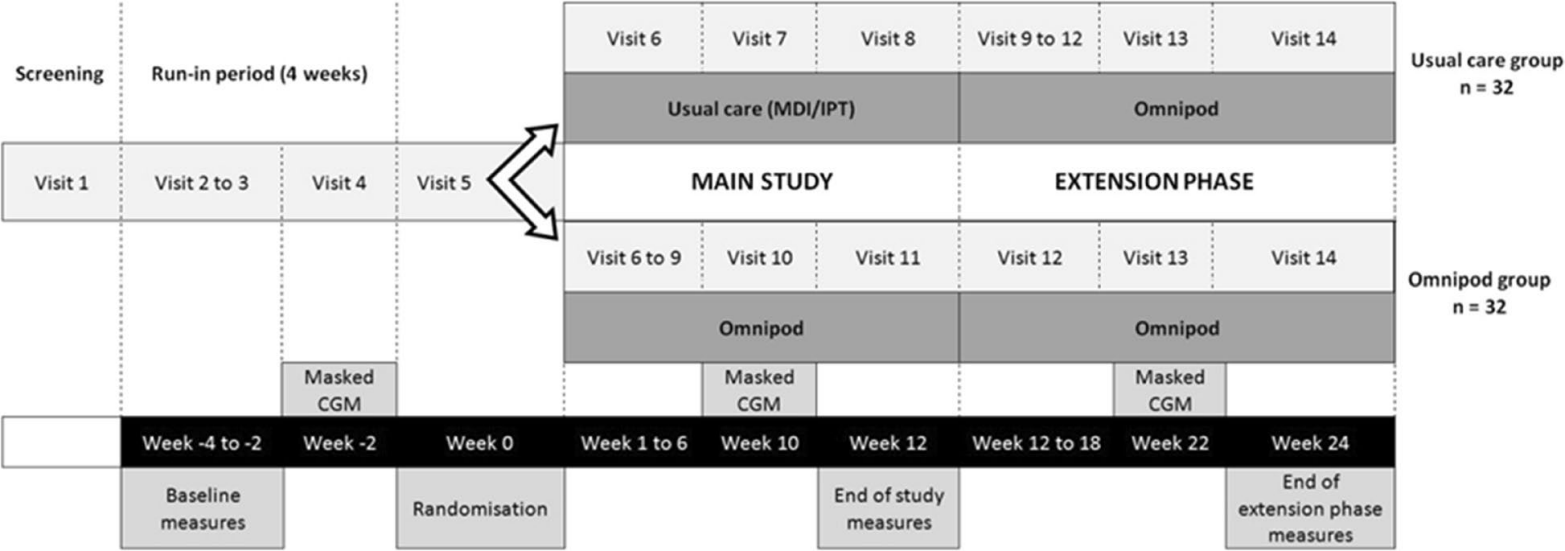
Study design summary

The trial (ACTRN12621001195842) is a 12-week exploratory multi-site parallel unblinded randomised controlled study, comparing treatment satisfaction with Omnipod DASH^®^ System vs. IPT using conventional insulin pumps or MDI on a background of glucose monitoring by SMBG. Allocation to intervention and control arms will be in a 1:1 ratio followed by an extension phase where all participants will be offered the Omnipod DASH^®^ System for 12 weeks (Fig. 2).

Progression to a large scale multi-site randomised controlled study will require demonstration that the study design is feasible with adequate study completion rates and that Omnipod DASH^®^ System is an acceptable intervention (if not more acceptable) than current modes of insulin delivery (MDI and conventional tubed IPT).

## Methods: participants, interventions and outcomes

### Study setting

The study will be conducted within Australia at four diabetes centres with St Vincent’s Hospital Melbourne as the lead site. Three of the study sites are metropolitan tertiary hospitals and academic centres (St Vincent’s Hospital Melbourne, Austin Health, Royal Melbourne Hospital), and one is a private regional specialist diabetes service (Geelong Endocrinology and Diabetes [Geelong]). All four participating centres have the physical infrastructure and human resources for the initiation and follow-up of patients with T1D on IPT.

### Eligibility criteria

All participants will provide written, informed consent prior to any study procedures occurring (See Additional file 1: Appendix 2 for sample informed consent form).

Full participant inclusion and exclusion criteria for study enrolment are listed in Table 1. At least 60% of participants recruited will be on MDI, reflecting the fact that the majority of adults with T1D in Australia are managed with injections [7]. The age range for inclusion of 18 to 70 years reflects 97.6% of T1D adults on IPT in the country [7]. Children with T1D are excluded because their diabetes technology user experience involves their caregivers and has a different focus.

**Table 1:** Eligibility

Inclusion criteria	Exclusion criteria
• Type 1 diabetes (as defined by the American Diabetes Association) [8] for at least 6 months • Insulin regimen consisting of either MDI or IPT • Age 18–70 years inclusive • HbA_1c_ < 10.0% (86 mmol/mol) • Willing to perform at least four SMBG readings daily • Willing to learn and apply carbohydrate counting skills • Willing to use Omnipod DASH^®^ Insulin Management System • Access to a computer with internet access • Proficient in English • Suitable for remote education	• Current use of real-time CGM (defined as the use of real-time CGM > 25% of the time during the past 3 months, except Abbott Libre™ 1 ISGM) • Use of any non-insulin glucose-lowering agent within the past 3 months • Oral or injected corticosteroid use within the past 3 months • An insulin requirement of more than 200 units of insulin every 2 days • An episode of severe hypoglycaemia or diabetic ketoacidosis within the past 3 months • Physical or intellectual disability precluding the use of IPT • Severe renal impairment (eGFR < 15 mL/min/1.73m^2^) • Haemoglobinopathy or haemolytic anaemia due to its interference with HbA1c assays • Major life-threatening illness impacting immediate life expectancy • Pregnancy, or planned pregnancy within the study period • Uncontrolled thyroid disease • Uncontrolled coeliac disease • Uncontrolled hypertension

Glucose monitoring will be by SMBG given that access to continuous glucose monitoring (CGM) for people with T1D varies from country to country depending upon social advantage and wealth. Prior to the 1st of July 2022, the CGM subsidy in Australia was restricted to people with T1D under 21 years old [9], with the majority of T1D adults performing SMBG readings due to the lack of CGM affordability. For this study, CGM use for less than 25% of the time during 3 months before study commencement is permissible based on previous evidence demonstrating no benefit in glycaemic management with intermittent CGM use in T1D [10]. The use of intermittently scanned glucose monitoring (ISGM) without hypoglycaemia/hyperglycaemia alarms (Abbott Libre™ 1) is also permissible because previous data demonstrated that ISGM without alarms does not equate to real-time CGM as it is not associated with improvements in HbA_1c_ or time in range compared to SMBG in T1D [11, 12]. The Abbott Libre™ 1 ISGM does not have the ability to show real-time glucose data unless scanned and does not have predictive high or low alert alarms, so users are not automatically prompted to change treatment decisions [11]. Participants using Libre™ 1 ISGM must be willing to perform at least four SMBG readings daily for study inclusion.

Participants are restricted to those with a maximum insulin requirement of 200 units every 2 days because the Omnipod DASH^®^ System is worn for 2 to 3 days and can only hold 200 units of insulin [4]. Therefore, those with a higher insulin requirement than this are not suitable.

### Interventions

All eligible participants will receive a refresher in general diabetes education and carbohydrate counting during a 4-week run-in to minimise the impact of diabetes education as a confounder when assessing outcomes. Participants will then be randomised in equal proportions between intervention (Omnipod DASH^®^ Insulin Management System) and usual care where they will continue to use their own insulin delivery method, for 12 weeks. Prior to the provision of the Omnipod DASH^®^ Insulin Management System, a member of the study team will calculate and enter the insulin delivery settings in the participant’s study device, and they will be trained in all aspects of its operation. Following the initiation of Pod therapy participants will have study follow-up appointments for insulin dosing adjustment which have been scheduled to reflect clinical practice. At the end of the 12-week study, an extension phase will be implemented where those allocated to usual care will be provided with the opportunity to receive the intervention and those who are on the intervention can continue with the device for a further 12 weeks.

### Modifications

Discontinuation of the intervention may be initiated at the request of the participant or if there are any concerns on the part of the investigation team regarding the safety of the participant e.g. the person is unable to operate the insulin delivery device safely despite remedial action with evidence of severe and persistent hyperglycaemia and/or ketosis or recurrent hypoglycaemia. Under these circumstances, the study team will cease therapy with Omnipod DASH^®^ System and ensure the participant’s safe transition to their usual mode of insulin delivery.

### Adherence

To maximise adherence to the protocol, a formal checklist is used during the consenting process to ensure that the participants fully understand what will be required of them when they enter the study. The requirement to complete the general diabetes education and carbohydrate counting training during run-ins will also help to ensure that only those who are able to fulfil the requirements of the protocol are randomised. Participants allocated to intervention will be educated in the use of the device by a skilled trainer which will help to ensure that the devices are used optimally. Follow-up of all participants, including review of glucose meter and device uploads, will be done by a clinically experienced team of doctors and diabetes nurse educators ensuring that the participants feel supported during the study and meet the requirements of the protocol. In addition, prior to study appointments participants will receive an automatically generated text message reminder.

### Concomitant care

In both intervention and usual care study arms, changes in insulin dosing can be made in response to the study team’s assessment of the participant’s glucose control. All other aspects of the participant’s care (e.g. antihypertensive, lipid-lowering, antidepressant medications) are to be continued throughout the study with changes to regimens decided upon according to clinical review by their usual health care professionals.

### Participant timeline

**Fig. 3:**
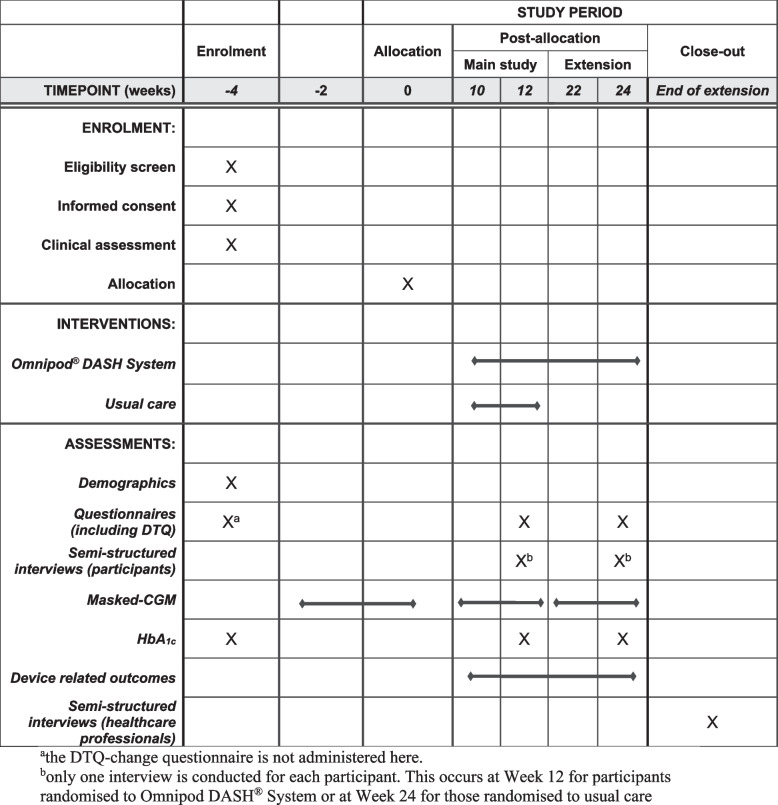
SPIRIT Figure summarising the study design

The study consists of a total of 14 visits during the 4-week run-in (weeks 4 to 0), the 12-week randomised controlled study (weeks 0 to 12) and the extension period (weeks 0 to 24) (Fig. 3). Some study visits may be conducted remotely at the investigators’ discretion following experience with the COVID-19 pandemic. If required additional contact with the study team will be available. Investigators will document communications and additional reviews with participants in the participant file.

**Table 2:** Study outcomes

Primary feasibility outcomes	
Acceptability of Omnipod DASH^®^ System vs. usual care	Difference in Diabetes Technology Questionnaire ‘current’ (DTQ-current) score between baseline and 12 weeks post-randomisation in that proportion of participants who complete the study
Secondary feasibility outcomes	
User acceptance	Diabetes technology treatment satisfaction: a) DTQ ‘current’ at 24 weeks post-randomisation b) DTQ ‘change’ at 12 and 24 weeks post-randomisation
Process outcomes	a) Recruitment and retention rates b) Frequency of additional study visits required c) Clarity of the inclusion and exclusion criteria as assessed feedback from the trial teams
Resource outcomes	a) Time taken for study procedures (e.g. education on the use of Omnipod DASH^®^ System) b) Mode of interaction (face-to-face vs. virtual) c) Device-related outcomes i. Length of time taken to change Omnipod DASH^®^ pods vs. insulin pump infusion sets (for participants on IPT) ii. Length of time taken to upload Omnipod DASH^®^ PDM data vs. pump data (for participants on IPT) iii. Frequency of Omnipod DASH^®^ pod failures vs. insulin infusion line occlusions (for participants on IPT) *Pre-defined as:* • *Unexplained finger-prick glucose reading* > *14 mmol/L that does not respond to a corrective dose of insulin with* ≥ *2.8 mmol/L drop within one hour* • *Finger-prick glucose reading* > *14 mmol/L with elevated ketones* ≥ *0.6 mmol/L* • *Occurrence of a non-resolvable pump occlusion/failure alarm)*
Secondary scientific outcomes	
Participant-centred outcomes	Questionnaires: a) Diabetes distress: Problem Areas in Diabetes (PAID) questionnaire b) Sleep quality: Pittsburgh Sleep Quality Index (PSQI) c) Fear of hypoglycaemia: Hypoglycaemia Fear Survey Short-Form (HFS-II-SF) d) Diabetes treatment satisfaction: Diabetes Medication System Rating Questionnaire Short-Form (DMSRQ-SF) e) User satisfaction: User Evaluation Questionnaire (UEQ) f) Treatment system efficacy and effectiveness: System Usability Scale (SUS)
	Participant perceptions of Omnipod DASH^®^ System assessed via semi-structured interviews
Healthcare professional perspectives	Healthcare professional perceptions of Omnipod DASH^®^ System assessed via semi-structured interviews
Glycaemic outcomes	Masked CGM metrics a) Percentage of time spent < 2.8 mmol/L b) Percentage of time spent < 3.3 mmol/L c) Percentage of time spent < 3.9 mmol/L d) Percentage of time spent 3.9–7.8 mmol/L e) Percentage of time spent 3.9–10 mmol/L f) Percentage of time spent > 10.0 mmol/L g) Percentage of time spent > 13.9 mmol/L h) Percentage of time spent > 16.7 mmol/L i) Standard deviation and coefficient of variation of CGM j) Mean CGM glucose
	HbA_1c_
	Symptomatic hypoglycaemic events recorded in real time through KeyLead Health™ phone application *Defined as typical symptoms of hypoglycaemia with finger-prick glucose reading* < *3.9 mmol/L*

#### Screening and run-in period

Participants who pass screening will undergo a 4-week run-during which they will be provided with carbohydrate counting education by a specialist dietitian and trained on the study BG meter with a meal insulin dosing calculator during a face-to-face visit with the study dietitian. As proficiency in carbohydrate counting is a cornerstone of diabetes management [4], all participants, including those participants already on conventional insulin pumps, will not proceed to randomisation unless this has been demonstrated. If necessary, the run-in can be extended by 2 weeks to ensure proficiency.

Following carbohydrate counting education, all participants will then undergo 2 weeks of masked-CGM prior to randomisation. They will also receive education and written information on the use of the KeyLead Health™ mobile application used for data collection.

#### Post-randomisation

Participants randomised to Omnipod DASH^®^ System will upload data from the study meter and PDM at clinic reviews in weeks 1, 2, 3 and 6. These reviews may be performed remotely at the discretion of the study team. During clinic reviews, all participants will have diabetes therapy adjustments (PDM settings for Omnipod DASH^®^ System or insulin doses for MDI/IPT) as necessary in conjunction with study clinicians. Participants randomised to usual care will continue using their usual insulin delivery modality (MDI or IPT). They will upload data from the study meter (and insulin pump if on IPT) at clinic review in week 6.

#### Extension phase

After 12 weeks, all participants will be provided with the opportunity to take part in the extension phase. Those on usual care will transition to the Omnipod DASH^®^ System and upload data from the study meter and PDM at clinic reviews in weeks 13, 14, 15 and 18 for adjustments of insulin dosing. These reviews may be performed remotely at the discretion of the study team. Those assigned to the intervention arm will continue using the Omnipod DASH^®^ System during the extension phase and upload device data at clinic review in week 18.

Data to be collected from all participants at the end of the study (week 12) and at the end of the extension phase (week 24) will include 2 weeks of masked CGM which will be obtained from weeks 10 to 12 and weeks 22 to 24. At weeks 12 and 24, HbA_1c_, questionnaire responses and data from all study devices will also be collected. Semi-structured interviews will be undertaken at week 12 for participants randomised to Omnipod DASH^®^ System and at week 24 for participants randomised to usual care.

### Outcomes (Table 2)

#### Primary feasibility outcomes

These outcomes will inform power calculations for the future main study.

(i) Feasibility will be determined by the proportion of the number of participants (*n* = 64) who complete the study per-protocol at week 12. The study design will be deemed feasible if the proportion completing the study is at least 0.80.
(ii) Acceptability of the Omnipod DASH^®^ Insulin Management System in those who complete the study will be assessed by the change in DTQ-current score [13, 14] from baseline to week 12, with comparisons made with usual care. DTQ is a 30-item validated measure of satisfaction with diabetes technological tools used in T1D management. It yields a separate score for ‘current’ satisfaction (DTQ-current: How much is this a problem now?) and for ‘change’ in satisfaction (DTQ-change: How has it changed compared to before the study?), ranging from 30 to 150 for each score [13, 14]. While there is currently no single universally accepted tool for assessing treatment satisfaction with diabetes technology devices this tool is simple to implement and does not impose a significant added burden upon the participant. It has been used in other studies evaluating novel diabetes technologies [6, 15].

#### Secondary feasibility outcomes

(i) *User acceptance:* Differences between the intervention and usual care study arms for DTQ-change score at week 12 will be analysed as will DTQ-current and DTQ-change at the completion of the extension period (week 24) [13, 14] to determine if these may offer greater resolution in delineating differences in user-perceptions (Table 3).
(ii) *Process outcomes:* These will include clarity and suitability of inclusion and exclusion criteria, recruitment and retention rates, the need for additional study visits and a better understanding of data collection tools and outcomes including unanticipated responses to questionnaires. Reasons for study non-participation, exclusion and study withdrawal will be documented to provide information about the suitability of inclusion and exclusion criteria. If it is taking longer than 18 months for the completion of the recruitment of 64 participants, study inclusion and exclusion criteria may need to be reconsidered. The need for additional study visits will be documented on REDCap^®^. Revision to the study design will need to be considered if additional study visits are required by more than 20% of participants. The protocol does allow for remote education if a global pandemic restricts participant visits to the clinical trial centre. Data for the mode of interaction (face-to-face or virtual) will be documented on REDCap^®^. If relevant, the impact of face-to-face vs. remote education on outcomes will be explored.
(iii) *Resource outcomes:* These will include assessing the time taken to implement study procedures (e.g. education on the use of Omnipod DASH^®^ System) and determining whether the data collection tools selected (KeyLead™ and REDCap^®^) are appropriate and if so optimisation of their implementation. Data for the time taken for each study visit will be documented on REDCap^®^. Participants will be asked open-ended questions about their involvement with the study procedures. Although no formal qualitative analysis will be conducted, this will further inform the feasibility and acceptability of study procedures and identify possible areas of improvement. Device-related outcomes that provide information about the performance of Omnipod DASH^®^ System will be assessed, including the length of time taken for an action to be completed such as changing insulin pump infusion sets (for participants on IPT), changing Omnipod DASH^®^ Pods and uploading pump data for IPT and Omnipod DASH^®^ System. The frequency of insulin infusion line failures (for those on conventional insulin pumps) and Omnipod DASH^®^ System failures will also be recorded in real-time (Table 4). A failure is pre-defined as an unexplained BG of more than 14mmol/L that does not reduce by at least 2.8mmol/L within 1 h after a corrective insulin dose, or a BG of more than 14mmol/L with elevated ketones of at least 0.6mmol/L, or an occurrence of a non-resolvable pump occlusion/failure alarm [16]. Device-related outcomes will be recorded in real-time using the KeyLead Health™ phone application to minimise recollection bias

**Table 3:** Analysis of questionnaires

Questionnaire	Method of analysis	Score associated with more positive outcomes
Diabetes Technology Questionnaire (DTQ) ‘current’ (30 items) [13, 14]	Mean of the total score (out of 150) Mean score for each question (out of 5)	Higher
Diabetes Technology Questionnaire (DTQ) 'change' (30 items) [13, 14]	Mean of the total score (out of 150) Mean score for each question (out of 5)	Higher
Problem Areas in Diabetes (PAID) questionnaire (20 items) [6]	Mean of the total score (out of 100)	Lower
Pittsburgh Sleep Quality Index (PSQI) (19 items) [15]	Mean of the total score (out of 21) Mean score for each section: • Sleep quality • Sleep latency • Sleep duration • Habitual sleep efficacy • Sleep disturbance • Use of sleeping medication • Daytime dysfunction	Lower
Hypoglycaemia Fear Survey Short-Form (HFS-II-SF) (11 items) [15]	Mean of the total score (out of 44) Mean score for each subscale: • Behaviour (B) • Worry (W)	Lower
Diabetes Medication System Rating Questionnaire Short-Form (DMSRQ-SF) (20 items) [17]	Mean of the total score (out of 72) Mean score for each section: • Convenience satisfaction • Negative events • Interference • Self-monitoring of blood glucose burden • Efficacy • Social burden • Wellbeing • Treatment satisfaction • Treatment preference	Higher
User Evaluation Questionnaire (UEQ) (26 items) [18]	Mean of each scale: • Attractiveness • Perspicuity • Efficacy • Dependability • Stimulation • Novelty	Higher
System Usability Scale (SUS) (10 items) [19]	Mean of total score (out of 100)	Higher

**Table 4:** KeyLead Health™ phone application buttons/modules

Time-related study events	Frequency-related study events
Change pump set: length of time taken to change insulin pump infusion sets (for participants on IPT)	Insulin set failure: frequency of insulin infusion line occlusions (for participants on IPT)
Change pod: length of time taken to change Omnipod DASH^®^ Pods	Pod failure: frequency of Omnipod DASH^®^ pod failure
Upload: length of time taken to upload IPT and Omnipod DASH^®^ PDM data	Hypo event: frequency of symptomatic hypoglycaemia with BG level of < 3.9 mmol/L
	Other: frequency of any other events of concern for participants (e.g. skin reactions to pod adhesive)

#### Secondary participant-centred outcomes

Participant-reported outcomes for diabetes distress, sleep quality, fear of hypoglycaemia and diabetes treatment experience will be assessed through six validated questionnaires administered which have been employed in prior diabetes technology studies [6, 15, 17–19] at 12 and 24 weeks (Table 3). These include the Problem Areas in Diabetes (PAID) questionnaire [6], Pittsburgh Sleep Quality Index (PSQI) [15], Hypoglycaemia Fear Survey Short-Form (HFS-II-SF) [15], Diabetes Medication System Rating Questionnaire Short-Form (DMSRQ-SF) [17], User Evaluation Questionnaire (UEQ) [18] and System Usability Scale (SUS) [19]. The short forms of HFS-II and DMSRQ will be used to reduce participant burden, which will help to ensure that data collection is complete. Participants allocated to intervention will be interviewed regarding their experience with and their recommendations for further refinement of the Omnipod DASH^®^ System 12 weeks post-randomisation. The interview topic guide for participant interviews will be informed by literature regarding barriers, suboptimal use and discontinuation of IPT [4]. The interviews will be implemented by study nurses and audio-recorded at week 12 for participants randomised to Omnipod DASH^®^ System and at week 24 following the extension for those initially randomised to usual care. The interviews are expected to take approximately 20 min. The audio recordings will be professionally transcribed by a native English speaker for later thematic analysis using Braun and Clarke’s six-phase approach [20].

#### Secondary healthcare professional perceptions

The perceptions of the physicians and diabetes nurse educators involved in the conduct of the study will be assessed through interviews. Two healthcare professionals (one doctor and one nurse) from each of the four study sites will be interviewed about their perceptions of the Omnipod DASH^®^ System and their recommendations for further refinement at study completion. The topic guide for the healthcare professionals will be informed by analysis of the participant interviews and research that has explored clinician attitudes towards technology use [21, 22]. The interviews will be audio-recorded and transcribed for later thematic analysis [20].

#### Secondary glycaemic outcomes

Masked-CGM outcomes will be assessed using international consensus metrics [23, 24] allowing comparison with other studies. All participants will wear a standardised masked-CGM system (Abbott FreeStyle^®^ Libre™ Pro Flash Glucose Monitoring device [Abbott Diabetes Care, Alameda, California]) for 2 weeks pre-randomisation (week -2 to 0), at weeks 10 to 12 and at weeks 22 to 24. HbA_1c_ will be measured pre-randomisation, week 12, and week 24 in alignment with CGM data collection and assayed by a single DCCT-aligned laboratory. Comparisons will be made between study arms in week 12. A change in CGM time in the target range and change in HbA_1c_ of 5% and 0.3%, respectively, are clinically significant [6].

Hypoglycaemic events, defined as an event where typical symptoms of hypoglycaemia are accompanied by a finger-prick blood glucose (BG) measurement of less than 3.9mmol/L [16], will be recorded in real-time using the KeyLead Health™ (Melbourne, Australia) mobile application (Table 4). The replacement of paper diaries with an electronic platform is expected to improve adherence and accuracy (refer to Methods: Data Collection for details regarding KeyLead Health™).

### Sample size

The exploratory nature of the study precludes a power calculation because there is no prior randomised controlled study using Diabetes Technology Questionnaire ‘current’ (DTQ-current) score to assess treatment satisfaction with insulin patch pump devices. The sample size for this pilot study was determined as per time and resources. We anticipate that 64 participants with an estimated dropout of 10%, based upon prior experience with insulin pump studies, will provide exploratory data for assessment of primary feasibility outcomes for study completion rates and acceptability of the Omnipod DASH^®^ System device, and protocol implementation across clinical sites and insulin delivery modalities (MDI and IPT). Data collected in this pilot study will allow for the determination of sample size for a future full-scale randomised controlled study with an effect size of 80% power.

### Recruitment

Participants will be recruited from four major adult diabetes centres. Each clinical centre was chosen based on documentation for patient availability. The total pool of people with T1D attending these centres is approximately 3000. CGM uptake in the T1D population attending these centres is greater than in the general T1D population and estimated at approximately 50%. Nevertheless, this will provide a pool of about 1500 individuals from whom 64 will be recruited for the study. As insulin patch pumps are not currently in Australia as of study commencement and with the study extension all participants recruited will be provided with the opportunity to experience a novel insulin delivery platform, we expect that involvement in the study will appeal to the person living with T1D. With the uncertainties associated with the COVID-19 pandemic, we expect recruitment of the 64 participants to take approximately 18 months.

## Methods: assignment of interventions

### Allocation

All individuals who provide consent for participation and who fulfil the inclusion criteria will be randomly allocated to intervention or usual care in a 1:1 ratio using minimisation of two variables that are expected to be highly prognostic of the primary outcome. The variables are the study site (four clinical sites) and baseline insulin delivery modality (MDI/IPT). An employee of the University of Melbourne who is not a member of the study team at St Vincent’s hospital Melbourne will provide oversight of the randomisation process which will be performed upon request by the teams responsible for implementing the interventions using a computerised sequence generation via central randomisation software and implemented through an electronic participant record system. The clinical teams will be informed of study arm allocation by telephone.

### Blinding (masking)

The nature of the intervention does not allow for concealment of the allocation from either participants or investigators and therefore parameters determining emergency unblinding are not applicable. Outcome assessors (e.g. those calculating questionnaire scores) will be blinded to allocation status. Those responsible for data analysis will be aware of allocation status as this is inherent to the task.

## Methods: data collection, management and analysis

### Data collection methods

Personnel at each site will attend a start-up meeting where they will be trained in the study protocol and its requirements, data entry into the electronic-case record form (e-CRF) and the operation of study devices and platforms by which data will be uploaded and eliciting of information from study participants in a uniform reproducible manner during the scripted interviews.

#### The electronic-case-record form

REDCap^®^, a secure web application designed as a data management platform to support data collection for research studies, will be used to support the e-CRF. It allows for multi-site access over a secure web connection with authentication and data logging. Audit trails are maintained to track data manipulation and user activity. This platform is The Health Insurance Portability and Accountability Act (HIPAA) compliant.

For the purposes of this study, REDCap^®^ will be used to directly electronically input visit data via e-CRFs and questionnaire responses at all study sites. This will eliminate errors from paper form data transcription and minimise missing data.

#### Diabetes management software

Diasend^®^ by Glooko^®^ is an internet-based platform used to upload data from the Omnipod^®^ DASH PDM and the study blood glucose meter (CONTOUR^®^ NEXT ONE glucose meter). Participants can upload data from home through the downloaded Diasend^®^ Uploader software in their computer using a USB cable. The uploaded data is shared with the study site’s Diasend^®^ Clinic account. At study sites, device data is uploaded through the Diasend^®^ Transmitter.

#### Masked-continuous glucose data collection

The study masked-CGM (Abbott FreeStyle^®^ Libre™ Pro Flash Glucose Monitoring device) is a commercially available, disposable, minimally invasive, factory-calibrated CGM that does not require any user interaction after sensor insertion on the posterior upper arm. It automatically records 15-minutely glucose readings for 14 days, after which the CGM data is obtained by scanning the sensor with the reader device. The overall mean absolute relative difference (MARD) in a comparison of the system with a reference method (Yellow Springs Instrument, Yellow Springs, Ohio) is 12.3% [25, 26]. Data is uploaded from the device to LibreView which is Abbott’s proprietary internet-based platform used for data upload from the study CGM reader device.

#### KeyLead Health™ Platform

The study will employ this bespoke mobile- phone application to capture, manage and analyse participant experiences in real-time. Table 4 details the study data captured through the application. All information collected via mobile application will be transferred and stored securely in the diabetes clinical database. Participant confidentiality is always protected via adherence to the Australian Privacy Principles established by the Privacy Act 1988. Data is encrypted using the AES-256 standard at-rest and with TLS/SSL at-transit.

### Retention

Once a participant is recruited, the study team will make every effort to follow that participant for the duration of the study. Even though a participant may elect to discontinue using Omnipod DASH^®^ System, this does not necessarily imply that they have withdrawn from the study. They will be encouraged to remain engaged with follow-up with all outcome data collected which will be analysed according to intention to treat. As previously highlighted, a robust consenting process is important in ensuring participant retention. During the study, it will be reinforced to the participants as to why the study is being conducted and the importance to the diabetes community of the information being generated. All questions or concerns will be answered in detail in a timely manner with team members providing 24/7 support. Finally, participants will be offered the opportunity to participate in the extension study where all will use the patch pump which may incentivise retention.

### Data management

All data will be entered into the e-CRF electronically using the current generation University of Melbourne desktop computers on-line. The data entry screens will reflect the protocol. Modifications to data previously entered will be documented which will include a time stamp and identifier for the person entering the data. Weekly reports will be automatically generated regarding data missing from the e-CRF and reviewed by the lead site (DNO and KB) before forwarding site-specific reports regarding missing data, missing forms and missing visits to each study site. Study data are stored on the University of Melbourne server and backed up daily.

Hard copies of pathology results and other source documents will be identified by participant study number and stored in a locked cupboard in a room that can only be accessed by team members using a swipe card. It is an Australian requirement to store the data for 15 years.

### Statistical methods

Please refer to Additional file 1: Appendix 3 for the full statistical analysis plan. Feasibility will be confirmed if the proportion of participants completing the study is at least 0.80. Acceptability assessed by the change in the DTQ-current score between baseline and study end will be analysed on the basis of intention to treat using analysis of covariance (ANCOVA) with adjustment for baseline participant characteristics. Residuals will be explored to assess model fit. If model fit is poor, non-parametric analysis will be performed. Composite total scores and average of item responses for DTQ-current will be analysed. Cronbach’s alpha reliability coefficient will be calculated for DTQ-current and any other secondary outcome multi-item scale measure.

Comparison of user acceptance, secondary participant-reported and glycaemic outcomes between participants using Omnipod DASH^®^ System and continuing usual care will be performed. The analytical methods for DTQ-current will also be utilised for DTQ-change. Other continuous secondary participant-reported and glycaemic outcomes will be assessed through similar methods. Analysis of count outcomes will be performed through Poisson or negative binomial regression model and binary outcomes through a logistic regression model. Methods of analysis for questionnaires are listed in Table 3.

Resource outcomes will be assessed for normality using normal probability plots. Normally distributed continuous variables shall be presented as mean ± standard deviation (SD), and non-normally distributed continuous variables shall be presented as median and interquartile range (IQR) and categorical variables as total number and percentage (%).

Patient characteristics by the randomisation group will be compared using independent sample *t* tests for continuous variables, Pearson’s chi-square test for categorical variables and Wilcoxon rank-sum test for variables without a normal distribution. A *p* value of < 0.05 will be considered statistically significant.

Interview data will be analysed by thematic analysis [20]. Subgroup analysis by baseline insulin delivery modality will be performed in the form of an interaction term in the regression model or through a stratified analysis for non-parametric methods. Differences in pre-trial insulin delivery modality (IPT as compared to MDI) will be assessed using Pearson’s chi-square test as this may provide insights into any major differences in outcomes associated with baseline insulin delivery modality. However, we acknowledge the limitations of such an analysis based upon the small numbers in each subgroup.

All primary and secondary outcome results will be reported with no adjustment for multiple comparisons.

### Data monitoring

As the intervention (Omnipod DASH^®^ System) is approved by the Therapeutic Goods Administration of Australia and its use will be in accordance with this approval by centres’ experts in the care of people living with T1D a data monitoring committee will not be convened.

### Harms

Each investigator has the responsibility to ensure arrangements are in place to record, notify, assess, report, analyse and manage adverse events in this study to comply with the Therapeutic Goods Administration (TGA) regulations and local Ethics requirements. Standard operating procedures for reporting all adverse events, device-related adverse events and severe adverse events are in place. The HREC will be informed of any serious adverse events and any unexpected device-related adverse events.

In addition to site-specific reports, the chief investigator (DNO) will be notified of all potentially related serious adverse events immediately or within 24 h of being made aware of the event to ensure appropriate notification to the HREC at the lead site.

### Auditing

The study project manager (KB) employed by the lead site, but not responsible for the implementation of the protocol, will audit all data entry at each site once each month with study teams at each site to ensure that the study is carried out according to the protocol and to Good Clinical Practice (GCP) standards, with robust systems for reporting adverse events.

### Confidentiality

All personal information about potential and enrolled participants will be de-identified to protect confidentiality before, during and after the trial. All study-related documents will be stored securely in a locked filing cabinet onsite in a room accessed only by a swipe card. All study data and laboratory specimens will be identified by unique identification number. All records that contain names or other personal identifiers, such as locator forms and informed consent forms, will be stored separately from study records identified by code number. Electronic databases will be secured on a university server with password-protected access systems.

### Access to data

The chief investigator will have access to datasets at all participating sites. In addition, principal investigators at each site will have direct access to their own site’s data sets. Upon completion of the study, all principal investigators will be provided with the final dataset on a password-protected USB. Data accessed by members of the team who are not directly involved with participants will be deidentified.

### Ancillary and post-trial care

Participants enrolled in the study are covered by indemnity for negligent harm through the sponsoring institution’s (St Vincent’s Hospital Melbourne) insurance. This will include cover for additional health care, compensation or damages. Post-trial all participants will be transitioned back to their usual care and provided with a summary of their glucose levels and test results, a copy of which will be forwarded to their general practitioner and usual diabetes care team.

### Dissemination policy

Aggregate data from all study sites will be analysed and following review by all co-authors will be submitted to a peer-reviewed journal. At this stage, a single publication is envisaged. Authorship will be determined by contribution to the design and implementation of the study and composition and review of the manuscript. The results of the study will also be disseminated at national and international conferences.

## Discussion

Improvements in glucose control for people with diabetes, while important, lack meaning if therapy is not acceptable to the person living with this life-long condition. Therefore, participant-reported outcomes are increasingly recognised as being of significance and of relevance to real-world outcomes following the implementation of new diabetes technologies. Regarding this, the user interface is pivotal to user acceptance and patch pump technology offers the person living with T1D the option of a discreet, non-intrusive delivery platform. However, despite the availability of patch pump technology for over ten years, this pilot randomised control trial is the only study to date which has as its primary focus the acceptability of tubeless insulin pump therapy.

In line with this absence of data, no interview analysis has been published on the lived experiences of novel users of patch pump technology. This evidence gap is in stark contrast to the ever-increasing number of interview studies regarding diabetes technology [27–30]. It will also be the first study to explore attitudes towards insulin patch pump use from healthcare professionals, highlighting a need for more research regarding clinician attitudes towards diabetes technologies [21].

The comparator, MDI or IPT with a conventional tubed insulin pump, represents the two alternatives in insulin delivery in ambulatory diabetes care to a patch pump. As most adults with T1D in Australia use MDI it was predetermined that a minimum of 60% of those recruited would be using this insulin delivery modality. A sub-analysis will provide preliminary data regarding participant-reported outcomes according to each of the two insulin delivery modalities.

As the aim of this pilot study is to evaluate the feasibility and generate preliminary data for a randomised control study comparing the user acceptance of insulin delivery with the patch pump vs. usual care, we therefore deliberately excluded real-time-CGM use as it would represent a significant confounding factor. This is because real-time CGM may be either stand-alone with MDI or IPT or may be integrated as part of IPT with alarms only, low-glucose suspends or fully automated insulin delivery functionality. Given that this would have introduced a significant degree of heterogeneity, SMBG was decided upon to be the standard of glucose monitoring in each of the study arms, particularly because most people living with T1D in Australia and globally at the time the study design was finalised were monitoring their glucose levels with SMBG. While CGM data will be collected during the study, this information will be masked to the participants. Masking CGM information to participants will minimise its impact on participant behaviour and perceptions.

The carbohydrate counting and general diabetes education will help to ensure that the devices are used as intended and that variability in diabetes self-management skills does not influence differences observed between study arms.

Four trial sites that are experienced and resourced in the implementation of IPT have been selected to minimise variation in training and familiarity of the trial team with diabetes technology as a confounding factor. This includes a regional privatised service to ensure that the model of care was not restricted to metropolitan tertiary services. Variations in carbohydrate counting skills in participants could also impact perceptions of a new device. To minimise this influence, all participants underwent review and education in carbohydrate counting during the study run-in. In addition, our clinical experience in the initiation of IPT suggested to us that it takes approximately 3 months for a person to become comfortable with a new device and hence a 12-week intervention was selected. The extension to the main study would provide us with data over a longer period and would have the added benefit of incentivising participants to remain in the study.

The outcome measures selected are all validated tools. However, we acknowledge that there is no universally accepted method for assessing treatment satisfaction with diabetes technology devices. The primary outcome, DTQ-current, is simple to implement and does not impose a significant added burden upon the participant. The pilot study will provide the study team with insights into the performance and merits of these outcomes when reviewed relative to changes in other psychosocial parameters measured, and insights provided by the semi-structured interviews which allow for deeper exploration of their experiences and the perceived benefits and barriers with a patch pump.

In summary, this large pilot study will provide the first data regarding user acceptance of insulin patch pump technology in Australian T1D adults. We anticipate that this information will provide insight into the design (e.g. tools for assessment of participant-reported outcomes) of larger studies evaluating subjective outcomes which are of significance to the person living with T1D.

### Supplementary Information

**Additional file 1: Appendix 1.** Trial Registration Summary. **Appendix 2.** SVHM HREC Approved Participant Information and Consent Form. **Appendix 3.** Statistical Analysis Plan.

## Data Availability

Upon application, the de-identified dataset may be accessed for review or for post hoc analyses by members of the team or external parties may be performed upon application to the chief investigator and subject to the review and approval of all principal investigators. Any publications based on analyses not detailed in the trial registry will be declared as such in resulting publications.
