# Supplementary material for: A pilot randomised controlled parallel arm trial evaluating treatment satisfaction with the Omnipod DASH® Insulin Management System compared with usual care in adults with type 1 diabetes in Australia: rationale, study design and methodologies
Source: Pilot Feasibility Stud. 2023 Oct 9;9:171. doi: 10.1186/s40814-023-01400-4 (PMC10561483; doi:10.1186/s40814-023-01400-4)
Supplement: Supplementary file 1 — Additional file 1: Appendix 1. Trial Registration Summary. Appendix 2. SVHM HREC Approved Participant Information and Consent Form. Appendix 3. Statistical Analysis Plan. [file 40814_2023_1400_MOESM1_ESM.docx]

**Appendix 1:** Trial Registration Summary

| **Data category** | **Information** |
| --- | --- |
| Primary registry and trial identifying number | Australian New Zealand Clinical Trials Registry (ANZCTR)  ACTRN12621001195842 |
| Date of registration in primary registry | 8 September 2021 |
| Secondary identifying numbers | Nil |
| Source(s) of monetary or material support | Insulet Australia Pty Ltd |
| Primary sponsor | St Vincent’s Hospital Melbourne |
| Secondary sponsor(s) | Nil |
| Contact for public queries | *YWK, MD*  St Vincent’s Hospital Melbourne, Australia *+6143393020*  *kongyw@unimelb.edu.au* |
| Contact for scientific queries | *DNO, MD* St Vincent’s Hospital Melbourne, Australia |
| Public title | Comparison of Treatment Satisfaction between the Omnipod Insulin Pump and Usual Care in Adults with Type 1 Diabetes in Australia |
| Scientific title | A Pilot Study Comparing Treatment Satisfaction with the Omnipod Insulin Pump Compared with Usual Care in Adults with Type 1 Diabetes Living in Australia |
| Countries of recruitment | Australia |
| Health condition(s) or problem(s) studied | Type 1 diabetes |
| Intervention(s) | Active comparator: Omnipod DASH insulin pump for 24 weeks  Placebo comparator: Participants' usual diabetes management (either multiple daily insulin injections or insulin pump therapy) for 12 weeks, followed by 12-weeks of using Omnipod DASH insulin pump (total duration of 24 weeks) |
| Key inclusion and exclusion criteria | Ages eligible for study: 18 to 70 years Sexes eligible for study: both Accepts healthy volunteers: no  Inclusion criteria: clinical diagnosis of type 1 diabetes (as defined by the American Diabetes Association) of at least six months duration managed with multiple daily injections (MDI) or insulin pump therapy (IPT), age 18-70 years old, fluent in English (reading and writing), willing to perform at least four self-monitoring of blood glucose (SMBG) readings each day, willing to learn how to carbohydrate count and to apply this knowledge, HbA_1c_ <10.0%, willing to use an Omnipod DASH insulin pump, has a computer with internet access, has access to a smart device with data plan.  Exclusion criteria: using real time-CGM defined as use >25% of the time during the past 3 months (Abbott Libre allowed), an episode of severe hypoglycemia or diabetic ketoacidosis within the preceding three months, a physical or intellectual disability precluding the use of insulin pump therapy, severe renal impairment (eGFR less than 15 ml per minute per 1.73m^2^), haemoglobinopathy or hemolytic anemia due to its interference with HbA_1c_ assays, major life-threatening illness impacting immediate life expectancy, use of any non-insulin glucose-lowering agent within the past 3 months, oral or injected corticosteroid use within the past 3 months, pregnancy or pregnancy planned within study period, uncontrolled thyroid disease, celiac disease or hypertension, a requirement for more than 200 I.U. of insulin every two days. |
| Study type | Interventional Allocation: randomised Intervention model: other, participants will be randomised to continue their usual care or commence on the Omnipod DASH insulin pump at the start of the intervention (Week 0) for 12 weeks, then both arms will be on the Omnipod DASH insulin pump for another 12 weeks as an extension Masking: open (masking not used) Primary purpose: treatment |
| Date of first enrolment | 15 July 2021 |
| Target sample size | 64 |
| Recruitment status | Recruiting |
| Primary outcome(s) | Treatment satisfaction measured by the difference in the Diabetes Technology Questionnaire (DTQ) score (Week 12 post intervention commencement) |
| Key secondary outcomes | Psychological outcomes measured by the Problem Areas in Diabetes (PAID) questionnaire, Pittsburgh Sleep Quality Index (PSQI), Hypoglycemia Fear Short-Form Survey (HFS-11-SF) and Diabetes Medication System Rating Questionnaire Short-Form (DMSRQ-SF).  Effectiveness, efficiency and user satisfaction with Omnipod DASH insulin pump measured via User Experience Questionnaire (UEQ) and System Usability Scale (SUS).  Acceptability of Omnipod DASH insulin pump device assessed via an open-ended one-on-one interview.  Masked continuous glucose monitor (CGM) metrics including percentage of time in range (3.9-10 mmol/L), percentage of time <3.9 mmol/L, percentage of time >10 mmol/L, percentage of time >13.9 mmol/L, percentage of time <2.8 mmol/L and mean glucose.  HbA_1c_ via blood test.  Number of symptomatic hypoglycemic events defined as an event with typical symptoms of hypoglycemia accompanied by measured plasma glucose concentration of 3.9 mmol/L or less, recorded by KeyLead App.  Length of time to change pods, length of time to change insulin pump sets, number of insulin infusion line occlusions and pod failures.  Healthcare professional perception of Omnipod DASH insulin management system as assessed via an open-ended 30-minute interview. |

**Appendix 2: SVHM HREC Approved Participant Information and Consent Form**

**Appendix 3:** Statistical Analysis Plan

**Statistical Analysis Plan – A Pilot Study Comparing Treatment Satisfaction with the Omnipod^TM^ Insulin Pump Compared with Usual Care in Adults with Type 1 Diabetes Living in Australia.**

**Research Question**

To examine study feasibility and acceptability of Omnipod DASH^®^ System compared with usual care i.e. multiple daily injections (MDI) or conventional insulin pump therapy (IPT) in people living with type 1 diabetes who are monitoring their glucose levels on capillary blood using a glucose meter. Please refer to study protocol where all aspects of the study design are provided.

**Study Cohort**

Sixty-four adults with a diagnosis of type 1 diabetes managed with MDI or IPT will be recruited from four specialist diabetes centres in Victoria, Australia.

**
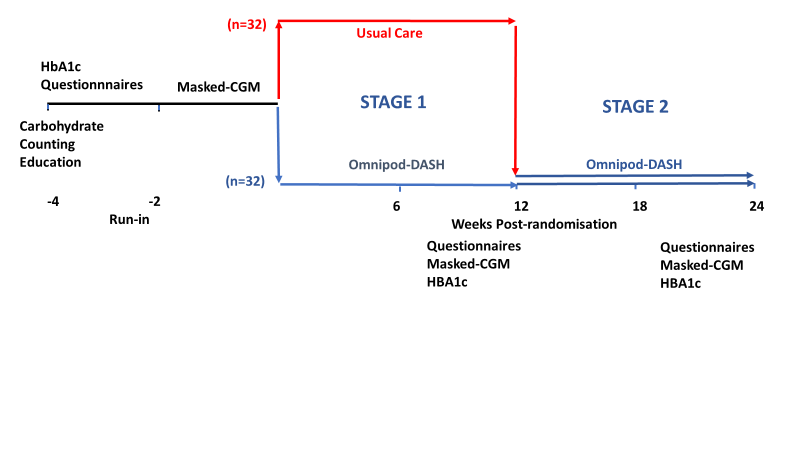
**

**Figure 1:** Overview of study design

**Primary Variables and Outcomes of Interest**

1. Acceptability of Omnipod DASH^®^ System compared with usual care assessed by the difference in Diabetes Technology Questionnaire (DTQ) “current” (DTQ-current) scores between baseline pre-randomisation and 12 weeks after the intervention in those participants completing the protocol via a Qualtrics survey (1, 2)

2. Feasibility of study completion as assessed by the proportion of participants who complete the study per-protocol. This will be defined as at least 0.80.

**Secondary Feasibility Endpoints**

User acceptance assessed via a Qualtrics survey including DTQ-current scores at 24 weeks; and DTQ-change scores at 12 weeks and 24 weeks (1, 2). Composite total scores for both DTQ change and current will be calculated by taking the average of item responses. Cronbach’s alpha reliability coefficient will be calculated for both versions (current and change) of the DTQ.

For process and resource outcomes, we will describe continuous data by summaries (e.g. means, medians, ranges) and categorical variables with frequency distributions. Outcomes, to be collected continuously in real-time using the study’s phone app, Keylead Health^TM^ platform, will include time duration and frequency of education, time to change pods, time to change insulin pump sets, and time to upload devices. Line occlusions (n) recorded by Keylead Health^TM^ platform, calls to help-lines (n), and skin reactions (n). A line occlusion will be predefined as 1) unexplained blood glucose >14mmol/L, not responsive to a corrective dose with at least a 2.8mmol/L drop within one hour, or 2) blood glucose >14mmol/L with ketones ≥ 0.6 mmol/L, or 4) occurrence of a non-resolvable pump occlusion alarm.

**Secondary Scientific Endpoints**

Participant reported endpoints also to be assessed via a Qualtrics survey including the Problem Areas in Diabetes (PAID) (3), Pittsburgh Sleep Quality Index (PSQI) (4), Hypoglycaemia Fear Survey – Short Form (HFS-II-SF) (4) and the Diabetes Medication System Rating Questionnaire – Short Form (DMSRQ-SF) (5).

At week 12 and 24 visits, the 26-item User Experience Questionnaire (UEQ) (6) and 10-item System Usability Scale (SUS) (7) will provide general measures of effectiveness, efficiency and user satisfaction with the Omnipod DASH^®^ insulin pump.

| Outcome - Questionnaire | Method of Scoring |
| --- | --- |
| Diabetes Technology Questionnaire (DTQ) Current Score | Mean of total score (out of 150)  Mean score for each question (out of 5) |
| Diabetes Technology Questionnaire (DTQ) Change score | Mean of total score (out of 150)  Mean score for each question (out of 5) |
| Problem Areas in Diabetes (PAID) | Mean of total score (out of 100) |
| Pittsburgh Sleep Quality Index (PSQI) | Mean of total score (out of 21)  Mean score for each section:   - Sleep quality - Sleep latency - Sleep duration - Habitual sleep efficacy - Sleep disturbance - Use of sleeping medication   Daytime dysfunction |
| Hypoglycaemia Fear Survey (short-form)(3) | Mean of total score (out of 44)  Mean score for each subscale: Behaviour (B), Worry (W) |
| Diabetes Medication System Rating Questionnaire – Short Form | Mean of total score (out of 72)  Mean score for each section:   - Convenience satisfaction - Negative events - Interference - Self-monitoring of blood glucose burden - Efficacy - Social burden - Wellbeing - Treatment satisfaction - Treatment preference |
| User Experience Questionnaire (UEQ) | Mean of each scale:   - Attractiveness - Perspicuity - Efficacy - Dependability - Stimulation - Novelty |
| System Usability Scale (SUS) | Mean of total score (out of 100) |

At the 12 and 24-week post-randomisation visit, participants using Omnipod DASH^®^ will be interviewed about the acceptability of the device and their recommendations for further refinement in implementation of the education. These interviews are expected to take approximately 20 to 30 minutes and will be audio recorded and transcribed for later analysis using Braun and Clarke’s reflexive thematic analysis approach (8).

Health Care Professionals (one from medical and nursing) from each site will be interviewed. These interviews are expected to take approximately 20 to 30 minutes and will be audio recorded and transcribed for later thematic analysis (8).

Glycaemic endpoints measured at the end of each stage will include CGM metrics standardized according to convention as described by Maahs *et al* (9) and HbA1c. Symptomatic hypoglycaemia, recorded on the Keylead Health^TM^ platform, is defined as an event during which typical symptoms of hypoglycaemia are accompanied by a measured plasma glucose concentration ≤70 mg/dl (3.9 mmol/l) and difference in the number hypoglycaemic events between the omnipod and standard care group will be examined.^6^

**Order of Statistical Analyses**

The exploratory nature of the study **precludes** a power calculation. N=64 participants will provide exploratory data across clinical sites and insulin delivery modalities (MDI and IPT).

The primary analyses will assess differences in the DTQ current score between the

Omnipod group and usual care at baseline and week 12 . This will be done on the basis of intention to treat using analysis of covariance (ANCOVA), with adjustment for baseline patient characteristics. A p-value of <0.05 will be considered statistically significant. Residuals will be explored to assess model fit. If model fit is poor, non-parametric analysis will be performed instead.

Continuous secondary outcomes will be assessed using ANCOVA, count outcomes will be assessed using a Poisson or negative binomial regression model and for binary outcomes, logistic regression models will be fitted.

A subgroup analysis will be conducted by baseline insulin delivery mode at 12 and 24 weeks in the form of an interaction term in the regression model or by stratifying analysis if non-parametric methods are required.

There will be no adjustment for multiple comparisons, all primary, secondary and tertiary outcome results will be reported.
